# SIRT1 Disruption in Human Fetal Hepatocytes Leads to Increased Accumulation of Glucose and Lipids

**DOI:** 10.1371/journal.pone.0149344

**Published:** 2016-02-18

**Authors:** Takamasa Tobita, Jorge Guzman-Lepe, Kazuki Takeishi, Toshimasa Nakao, Yang Wang, Fanying Meng, Chu-Xia Deng, Alexandra Collin de l’Hortet, Alejandro Soto-Gutierrez

**Affiliations:** 1 Department of Pathology, University of Pittsburgh, Pittsburgh, Pennsylvania, United States of America; 2 Thomas E Starzl Transplant Institute, University of Pittsburgh, Pittsburgh, Pennsylvania, United States of America; 3 McGowan Institute for Regenerative Medicine; Pittsburgh, Pennsylvania, United States of America; 4 Department of Hepatobiliary Surgery, Peking University People’s Hospital, Beijing, China; 5 Faculty of Health Sciences, University of Macau, Avenida da Universidade, Taipa, Macau, China; INRA, FRANCE

## Abstract

There are unprecedented epidemics of obesity, such as type II diabetes and non-alcoholic fatty liver diseases (NAFLD) in developed countries. A concerning percentage of American children are being affected by obesity and NAFLD. Studies have suggested that the maternal environment *in utero* might play a role in the development of these diseases later in life. In this study, we documented that inhibiting SIRT1 signaling in human fetal hepatocytes rapidly led to an increase in intracellular glucose and lipids levels. More importantly, both *de novo* lipogenesis and gluconeogenesis related genes were upregulated upon SIRT1 inhibition. The AKT/FOXO1 pathway, a major negative regulator of gluconeogenesis, was decreased in the human fetal hepatocytes inhibited for SIRT1, consistent with the higher level of gluconeogenesis. These results indicate that SIRT1 is an important regulator of lipid and carbohydrate metabolisms within human fetal hepatocytes, acting as an adaptive transcriptional response to environmental changes.

## Introduction

SIRT1 (Silent mating type information regulation 2 homolog) belongs to the Sirtuins family of proteins and codes for a class III NAD-dependent histone deacetylase (HDACs). In mammals, the functions of SIRT1 have been essentially linked to the regulation of growth, apoptosis, metabolism and aging, responding to environmental cues through NAD+ levels [1–4]. SIRT1 deacetylates a wide range of targets, leading to epigenetic modifications of histones and modulation of transcription factors or metabolic enzymes [3]. Thus, SIRT1 has been thought to be a molecular link between the adaptive transcriptional response and the metabolic status [5, 6]. Recent studies performed on the liver of mice showed the key role of SIRT1 in the development of fatty liver through the regulation of proteins involved in lipid [7, 8] and carbohydrate metabolism [9–11]. These studies have highlighted the potential therapeutic use of SIRT1 in hepatic steatosis.

In recent years, the incidence of non-alcoholic fatty liver diseases (NAFLD) has increased dramatically in developed countries, present in more than 30% of the population in the U.S. It is associated with obesity, insulin resistance, and type II diabetes, and it predisposes the liver to the development of chronic inflammation and oxidative stress [12]. Nonalcoholic steatohepatitis or NASH is the combination of fatty infiltration of hepatocytes with the presence of inflammation. Ballooning degeneration of hepatocytes and increased Mallory’s hyaline inclusions often manifests this injury. Progressive inflammation in both pediatric and adult NASH patients can lead to scarring or fibrosis of the liver and, in severe cases, even cirrhosis and hepatocellular carcinomas (HCC). More importantly, pediatric liver steatosis has increased dramatically in the last 10 years, affecting more than 10% of American children [13]. Pediatric NAFLD is strongly associated with obesity and insulin resistance and its pathogenesis is not yet fully understood [14]. Most worrying is the fact that cardiovascular morbidity in children and teenagers are associated with NAFLD. Thus, understanding the pathogenesis, risk factors, and natural history of fatty liver disease is much needed to prevent youth at risk.

The role played by the environment in the development of NAFLD is very important despite potential genetic susceptibilities. Nicholas Hales and David Barker have proposed the “Thrifty phenotype hypothesis”, which speculate that the maternal nutrition during development may lead to type 2 diabetes, obesity, and the metabolic syndrome in the offspring later in life [15]. There have been several demonstrations of this “fetal origin of adult disease” in rodent models, testing the effect of caloric restriction or high fat diet on the first and second generations of offspring [16–19]. However, only few indications of such demonstrations have been observed in human [20, 21], thus, when and how the human body becomes susceptible to this environment remains unknown. Herein, we have investigated the role of SIRT1 in the lipid and carbohydrate metabolisms of human fetal hepatocytes and showed that a short-span inhibition of this protein lead to an upregulation of *de novo* lipogenesis and gluconeogenesis pathways in human fetal hepatocytes.

## Material and Methods

### Fetal human hepatocytes isolation and culture

The de-identified tissues were obtained from Magee Women’s Hospital (Pittsburgh, PA) and the University of Washington Department of Pediatrics, Division of Genetic Medicine, Laboratory of Developmental Biology (Seattle, WA) after obtaining a written informed consent by a protocol approved by the Human Research Review Committee of the University of Pittsburgh (Honest broker approval number HB015 and HB000836). Human fetal hepatocytes were isolated from fetal livers (Table 1) obtained after the termination of pregnancy performed at 20–23 weeks of gestation. Primary human fetal hepatocytes were isolated by digesting the tissue in EMEM (Lonza, Walkersville, MD), which contains 0.5 mg/ml of collagenase (Type XI, Sigma-Aldrich, Saint-Louis MO, Cat. #C7657), on a lab shaker for 40 minutes. Viability was assessed by trypan blue exclusion test and was routinely >85%. Fetal hepatocytes were plated at a density of 1.3x10^5^ cells/cm^2^ on type I rat tail collagen coated 12 well plates (Corning, Corning, NY). Cells were cultured for 3 days with a DMEM medium (Gibco, Life Technologies, Carlsbad, CA, USA) containing 1X penstrep, 10^-7^M of insulin (Sigma-Aldrich, Saint-Louis, MO), and 5% bovine serum albumin (Gibco, Life Technologies, Carlsbad, CA, USA). The SIRT1 pharmacological inhibitor (Sirtinol) was purchased from Chayman Chemical (Ann Arbor, MI, USA) and was added to the cells at a concentration of 50uM every 24h from day 0 to 3.

**Table 1:** Donor Demographics.

Tissue ID	Gestational age	Sex	Viability
Fetal liver 1	20 weeks	Male	87%
Fetal liver 2	20 weeks	Female	88%
Fetal liver 3	21 weeks	Unknown	88%
Fetal liver 4	23 weeks	Female	88%
Fetal liver 5	21 weeks	Male	89%
Fetal liver 6	23 weeks	Female	91%
Fetal liver 7	21 weeks	Female	87%
Fetal liver 8	20 weeks	Male	88%

### RNA extraction, Reverse Transcription and quantitative Polymerase Chain Reactions

Total RNA from the cultured human fetal hepatocytes were extracted using the RNeasy mini kit (Qiagen, Valencia, CA, USA) using the manufacturer's protocol. RNA was quantified by a spectrophotometer to assess the chemical purity and concentration of RNA. 2ug of RNA was mixed with random hexamers (Invitrogen, Carlsbad, CA), heated to 70°C for 5 minutes, and cooled to 4°C. RNA was then reverse transcribed using the Superscript III First-Strand Synthesis System (Invitrogen, Carlsbad, CA), following the manufacturer's protocol. Subsequently, qPCR was performed using TaqMan Fast Advanced Master Mix (Life Technologies, CA, USA) with a StepOnePlus system (Applied Biosystems, Foster City, CA) with the primers from Life Technologies (Carlsbad, CA, USA) described in Table 2. Expression of specific genes was normalized to an internal control Hypoxanthine-guanine phosphoribosyltransferase (HPRT) mRNA expression.

**Table 2:** List Primers.

Gene Name	Gene Symbol	Reference (Life technologies)
*acetyl-CoA carboxylase alpha*	ACACA	Hs01046047_m1
*ELOVL fatty acid elongase 6*	ELOVL6	Hs00907564_m1
*fatty acid synthase*	FASN	Hs01005622_m1
*glucose-6-phosphatase*, *catalytic subunit*	G6PC	Hs02560787_s1
*hypoxanthine phosphoribosyltransferase 1*	HPRT1	Hs02800695_m1
*phosphoenolpyruvate carboxykinase 2*	PCK2	Hs00388934_m1
*stearoyl-CoA desaturase*	SCD	Hs01682761_m1
*sirtuin 1*	SIRT1	Hs01009006_m1
*sterol regulatory element binding transcription factor 1*	SREBF1	Hs01088691_m1
*acyl-CoA dehydrogenase*	ACADM	Hs00936580_m1
*acyl-CoA oxidase 1*	ACOX1	Hs01074241_m1
*ATP-binding cassette*, *sub-family A (ABC1)*, *member 1*	ABCA1	Hs01059118_m1

### Metabolic content measurement

Cultured human fetal hepatocytes were washed with PBS and fixed with 4% PFA for 20min. Cells were stained with HCS LipidTOX red neutral lipid stain and Hoechst 33342 (Sigma-Aldrich, Saint-Louis, MO) for 30 min following the manufacturer's protocol. Triglycerides were extracted from fetal hepatocytes by mixing 10% of NP-40 to the cell pellets and by incubating at 80°C for 5min. The triglycerides were measured with a Triglyceride Quantification colorimetric assay (Biovision, Milpitas, CA) according to the manufacturer's instructions. Glucose concentration levels were measured from fetal hepatocytes medium by using the glucose autokit (Wako Chemicals, Richmond, VA).

### Histological assessment

Cultured human fetal hepatocytes were washed with PBS and fixed with 4% PFA for 20min. Incubations with primary antibodies (1:100 mouse anti-SIRT1, (Santa Cruz Biotechnology #sc-74504), 1:100 rabbit anti-phospho Ser473-Akt (Cell signaling #4060) and 1:400 rabbit anti-phospho Ser256-FOXO1 (Abcam #ab26651)) were carried out overnight at 4°C. For the detection of the primary antibodies, secondary antibodies (1:250 Alexa Fluor 488 Donkey anti-Mouse IgG (Life technologies CA, USA #A-21202), and 1:250 Alexa Fluor 488 Goat anti-Rabbit IgG (Life technologies, CA, USA #A-11008)) were used followed by counterstaining with 0.2 ug/mL of Hoechst 33342 (Sigma-Aldrich, Saint-Louis, MO). Images were captured using a Zeiss microscope. Acquired images were processed and quantified using ImageJ 1.48v software.

### Western Blot

A total of 30 μg of protein was resolved on Tris-HCl precast gels (Bio-Rad Laboratories, Hercules, CA) by SDSPAGE analysis. The proteins were transferred to polyvinylidene difluoride membranes. Membranes were incubated overnight at 4°C with the following primary antibodies: Mouse anti-SIRT1 sc-74504, Rabbit anti-glyceraldehyde-3-phosphate dehydrogenase sc-2577 (Santa Cruz Biotechnology, Santa Cruz, CA), Rabbit Akt #4691S, Rabbit Phospho-Akt-ser473#4060 (Cell signaling, Beverly, MA), Rabbit anti-FOXO1 ab70382, Rabbit Phospho-FOXO1-Ser256 ab26651, Mouse TORC2 ab105932 (Abcam, Cambridge, MA). The signal was amplified either by an anti-mouse or anti-rabbit HRP-conjugated secondary antibody (Millipore, Billerica, MA). Proteins were revealed using the SuperSignal West Pico Chemiluminescent Substrate (Thermo Fisher Scientific, San Jose, CA).

### Statistical analyses

All statistical analyses were performed with Prism 6.0 Software (GraphPad Software, Inc., La Jolla, CA) using non-parametric Mann-Whitney test. Data were presented as mean ± SEM, with P < 0.05 considered as statistically significant.

## Results

### Inhibition of SIRT1 induces an increase in lipids and carbohydrates levels in human fetal hepatocytes

**Fig 1:**
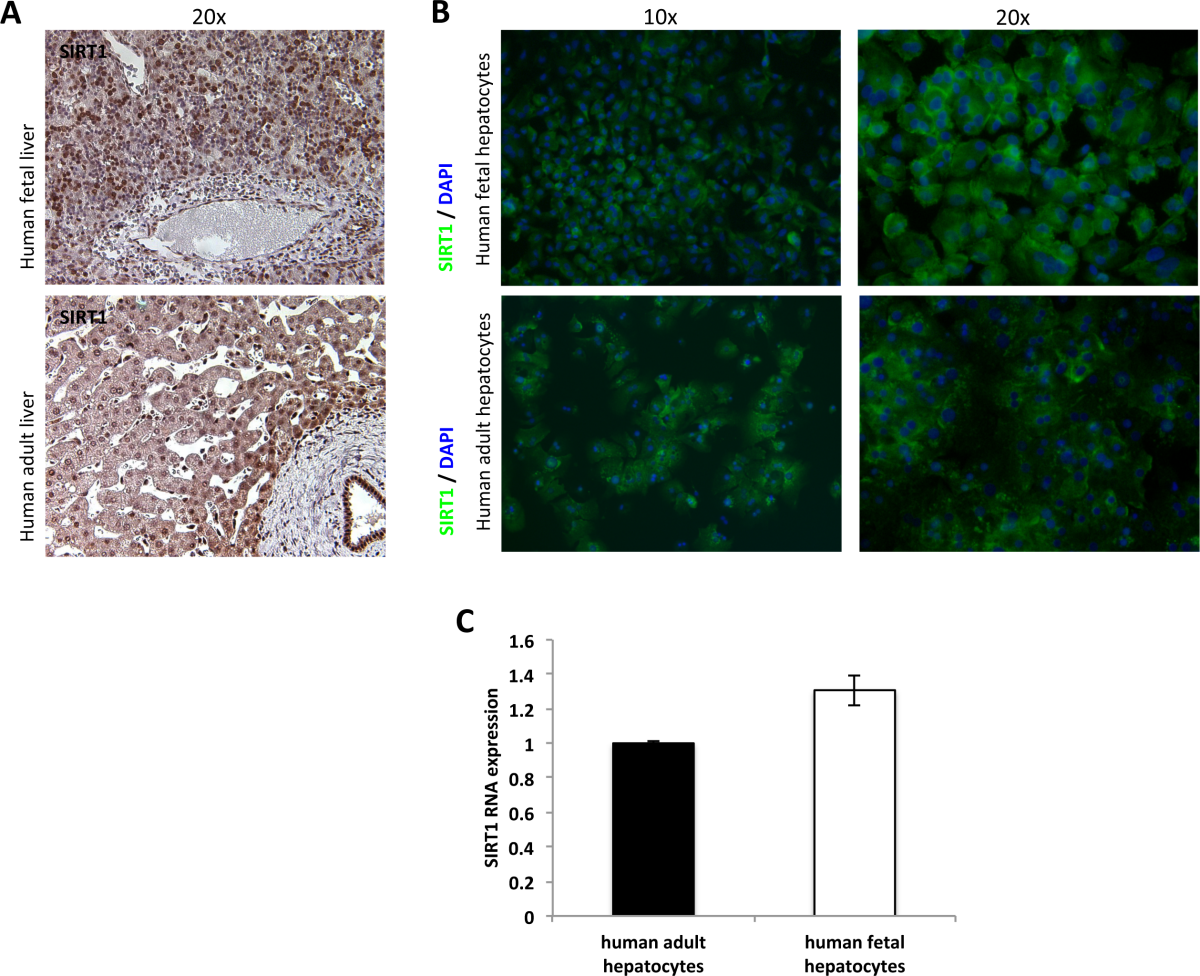
Expression of SIRT1 in human fetal and adult liver and isolated hepatocytes. (A) Immunohistochemistry of SIRT1 in human fetal and adult liver, (B) Immunofluorescence staining of SIRT1 in fetal and adult hepatocytes after 2 days of culture. (C) Quantification of hepatic SIRT1 mRNA expression measured by qRT- PCR in human fetal and adult hepatocytes in vitro and in vivo (n≥3/group).

There are numerous studies that address the role of SIRT1 in liver metabolism [8, 22–24]. In mice, it has been shown that the deletion of SIRT1 leads to hepatic steatosis with normal and high fat diet [7, 22]. Moreover, mice models of hepatic steatosis display low levels of SIRT1 [25]. Both the metabolism of carbohydrates and lipids were found to be upregulated in these models. The expression of SIRT1 in adult liver has been described, however, SIRT1 expression and role in human fetal hepatocytes is unknown. Thus, we first assessed the expression of SIRT1 in human fetal hepatocytes. Immune-staining of SIRT1 in fetal and adult liver tissue demonstrated that SIRT1 is expressed in both tissues (Fig 1A). Immune-histological images of adult and fetal hepatocytes in culture also exhibited a clear expression of SIRT1, mainly cytoplasmic with some nuclear localization (Fig 1B). This was further confirmed by western blot analysis of SIRT1 in human fetal hepatocytes (S1 Fig) and with qPCR analysis of SIRT1 expression (Fig 1B). These results strongly indicate that SIRT1 is highly expressed during development in human fetal hepatocytes.

**Fig 2:**
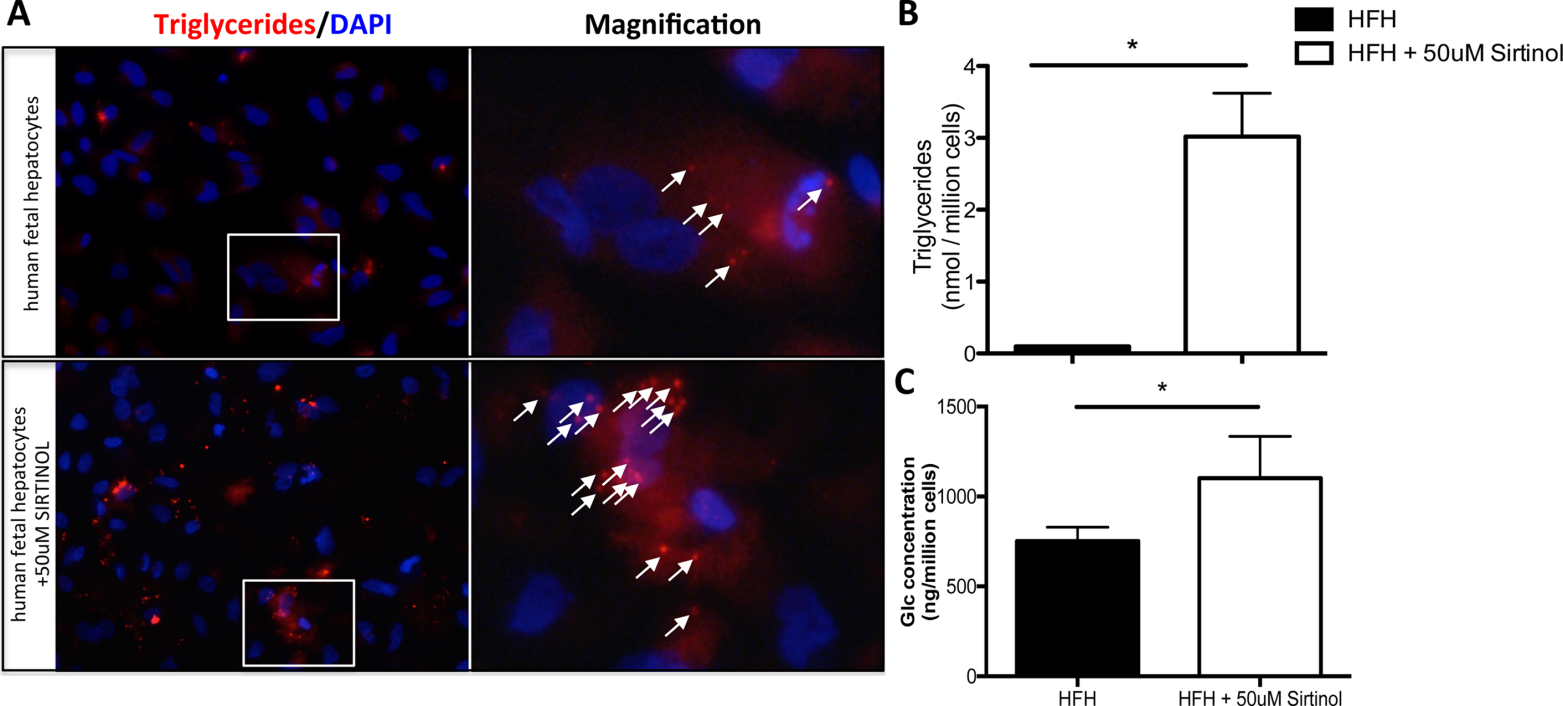
Lipids and glucose levels in human fetal hepatocytes after Sirt1 inhibition. (A) Fluorescent staining of lipids (red droplets, white arrow) inside human fetal hepatocytes (HFH) with or without 50uM Sirtinol for 3 days (20x). (B) Triglycerides quantification of human fetal hepatocytes with or without +50uM Sirtinol for 3 days analyzed by a colorimetric assay. (C) Glucose concentration between normal HFH and HFH +50uM Sirtinol for 3 days. (n≥7/group; *, P < .05).

To characterize the role of SIRT1 in lipid and glucose metabolism of human fetal hepatocytes, we used a pharmacological inhibitor of SIRT1, Sirtinol. Human fetal hepatocytes were exposed to 50uM of Sirtinol for three days. Intracellular lipid accumulation was analyzed using a fluorescent probe (Fig 2A). As expected, normal human fetal hepatocytes exhibit almost no intracellular lipid. However, we observed a clear increase in intracellular lipid accumulation when SIRT1 was inhibited. This was further confirmed by measuring the intracellular triglycerides. Human fetal hepatocytes exposed to Sirtinol showed a 30-fold increase in triglycerides accumulation compared to control (Fig 2B). We then measured the intracellular glucose levels. Human fetal hepatocytes inhibited for SIRT1 exhibited a significantly higher level of glucose concentration compared to control (Fig 2C). Altogether, these results showed that Sirtinol exposure leads to an increase in lipid and glucose levels in human fetal hepatocytes after SIRT1 inhibition.

### Inhibition of SIRT1 leads to an increased de novo lipogenesis in human fetal hepatocytes

**Fig 3:**
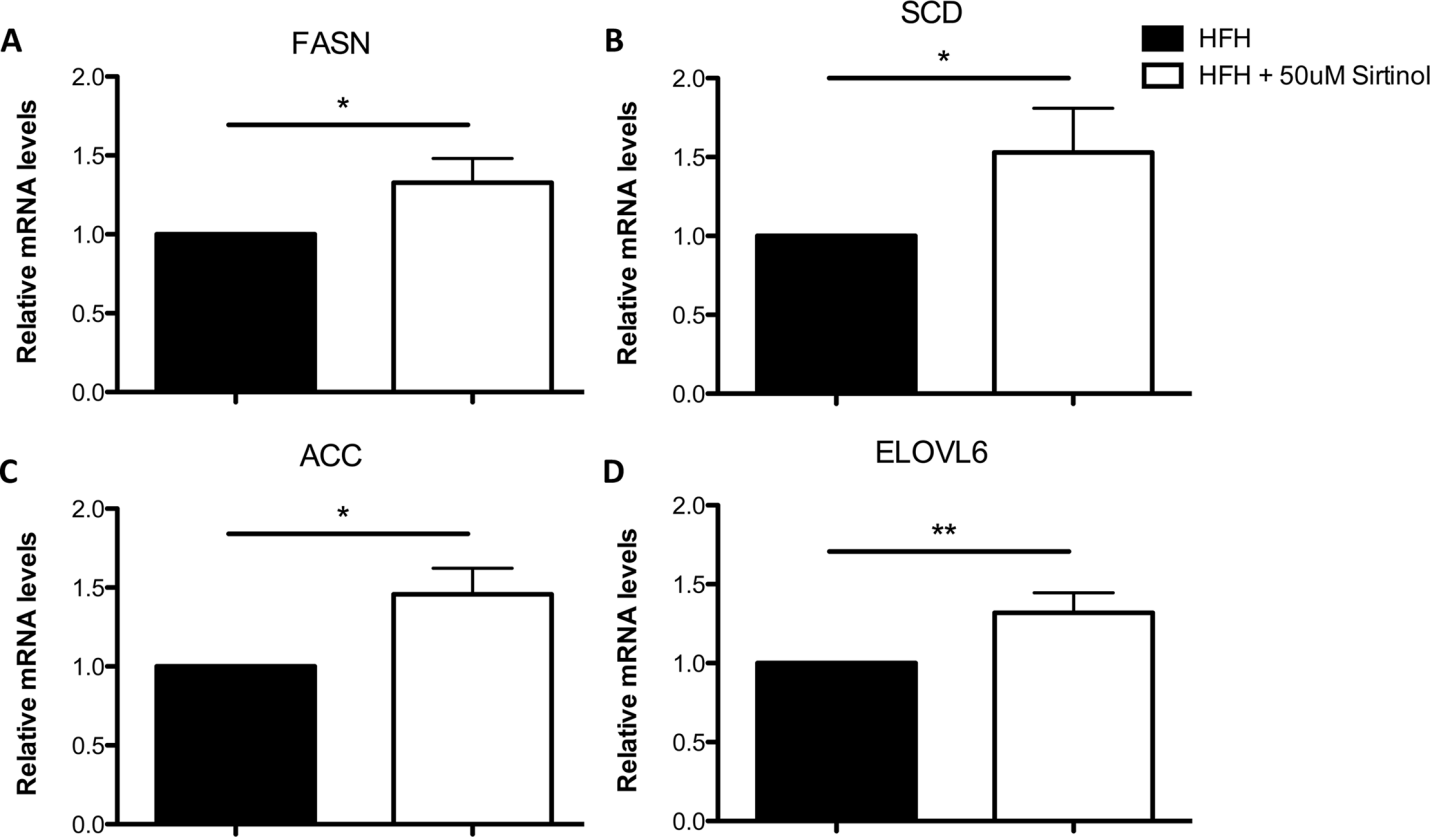
Increased activation of De Novo Lipogenesis related genes in human fetal hepatocytes after Sirt1 inhibition. Expression of hepatic FASN, SCD, ACC and ELOVL6 mRNA measured by qRT-PCR in human fetal hepatocytes exposed to +50uM Sirtinol compared to controls. (N≥7/group; *, P < .05)

Two potential pathways could explain the increase in lipid content after SIRT1 inhibition: a decrease in β-oxidation and increase in *de novo* lipogenesis pathway. We examined the mRNA levels of two gene targets of β-oxidation, acyl-CoA dehydrogenase (ACADM) and acyl-CoA oxidase 1 (ACOX1). We could not detect any difference between human fetal hepatocytes with or without Sirtinol (S2A Fig). This can be explained by previous findings reporting that β-oxidation of fetal livers is low. Shortly after birth, exposure of fatty acids through the mother’s milk induces the activation β-oxidation through CPT1-a [26]. Moreover, SIRT1 liver inhibition in rodents has been shown to induce an increase in *de novo* lipogenesis, leading to hepatic steatosis [7]. Thus, we studied genes involved in *de novo* lipogenesis by performing qPCR 24h after Sirtinol exposure. We found that all four major genes involved in *de novo*—fatty acid synthase (FAS), acetyl-CoA carboxylase (ACC), stearoyl-CoA desaturase (SCD), and elongase of long chain fatty acids family 6 (ELOVL6)—were upregulated when the cells were exposed to SIRT1 inhibitor (Fig 3A, 3B, 3C and 3D). These findings are very consistent with previous published data showing an increase in *de novo* lipogenesis associated with SIRT1 inhibition in rodents [7] and explain the rapid increase in lipid production and accumulation of human fetal hepatocytes.

### Inhibition of SIRT1 leads to an increased gluconeogenesis in human fetal hepatocytes through the impairment of AKT/FOXO1 pathway

**Fig 4:**
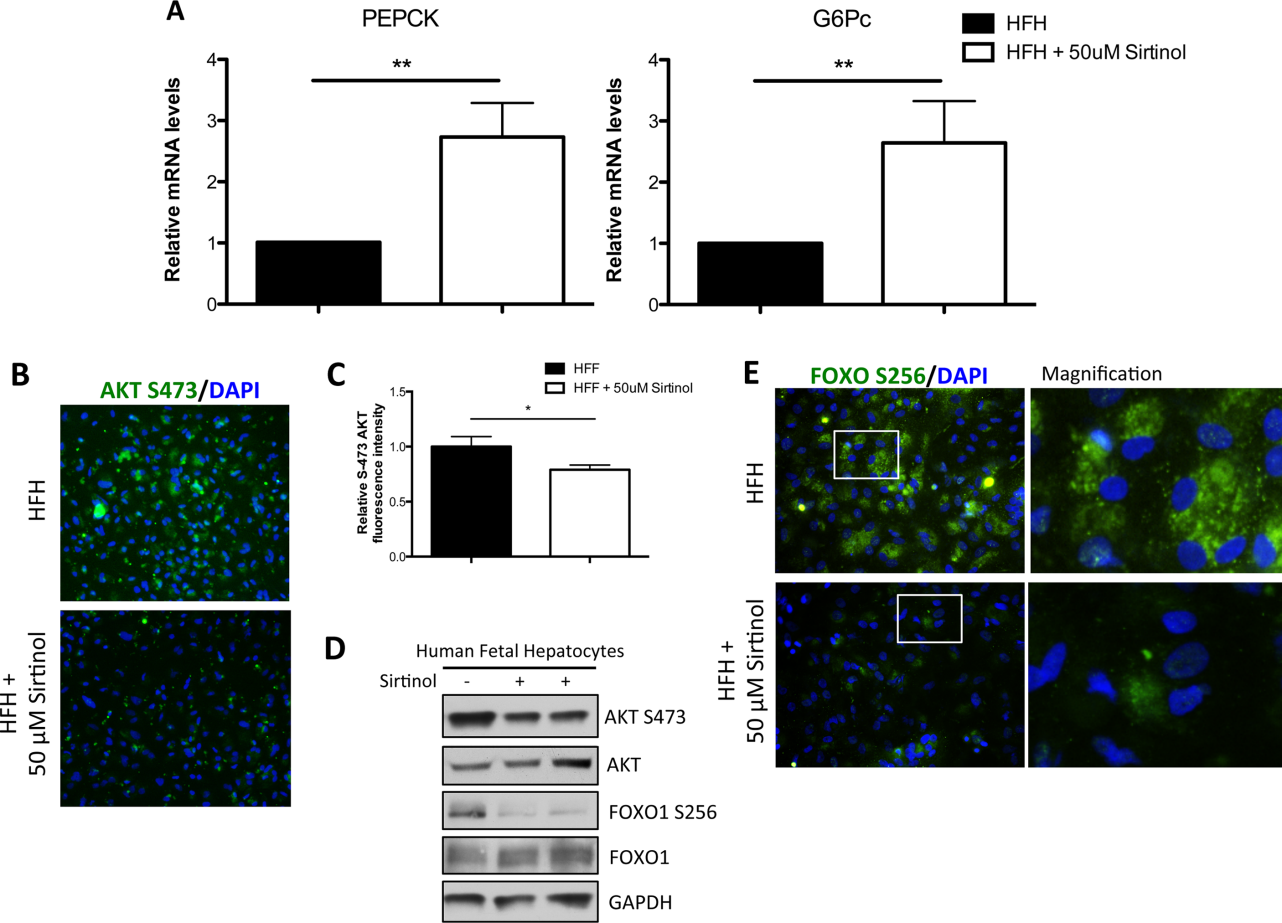
Increased activation of Glucogenesis pathway in human fetal hepatocytes after exposure to Sirtinol. (A) Expression of hepatic PEPCK and G6PC mRNA measured by qRT- PCR in human fetal hepatocytes exposed to +50uM Sirtinol compared to controls. (B) Immunofluorescence of S-473 AKT in human fetal hepatocytes with or without 50uM Sirtinol (10x). (C) Fluorescence intensity quantification for S-473 AKT signal (arbitrary units) using ImageJ (n≥7/group; *, P < .05). (D) Western blot analysis for S-473 AKT/AKT, S-256 FOXO1/FOXO1 in human fetal hepatocytes exposed to 50uM Sirtinol compared to controls. GAPDH was used as a loading control. (E) Immunofluorescence staining of S-256 FOXO1 in human fetal hepatocytes with or without 50uM Sirtinol (20x).

Previous studies have reported controversial results regarding changes in glucose production and use in rodent models with SIRT1-knockout [10, 11]. More recently, we reported that rodents carrying a liver-SIRT1 knockout exhibited hyperglycemia and had an increase in gluconeogenesis, which lead to insulin resistance [9]. Herein, we first investigated gluconeogenesis in human fetal hepatocytes by measuring the transcript of two main gluconeogenetic enzymes: phosphoenolpyruvate carboxykinase (PEPCK) and the catalytic subunit of glucose-6-phosphatase (G6Pc). Both exhibited approximately 3-fold increases, which was consistent with the increase in glucose concentration observed (Fig 4A). Inside the liver, the gluconeogenesis pathway is known to be under the control of AKT/FOXO1 [27]. Forkhead box O1 (FOXO1) usually enters the nucleus and eventually leads to the activation of genes linked to gluconeogenesis. However, the activation of AKT induces the phosphorylation of FOXO1, which lead to the exclusion of this protein out of the nucleus to the cytoplasm. This system controls the level of gluconeogenesis inside the cell [27]. Thus, to further explain the upregulaltion of the gluconeogenesis pathway, we first checked the activation of AKT at serine 473 through immunofluorescence staining of human fetal hepatocytes. AKT phosphorylation was found in both cells, but to a lower extend when Sirtinol was present (Fig 4B and 4C). Western blots analysis corroborated the decrease of AKT-S473 activation in human fetal hepatocytes inhibited for SIRT1 (Fig 4D). We then measured the phosphorylation of FOXO1, downstream of AKT. FOXO1 S256 decreases in human fetal hepatocytes with Sirtinol, which indicate a higher FOXO1 activity inside the nucleus (Fig 4D). As expected, immunofluorescence staining of FOXO S256 exhibited a cytoplasmic localization of the protein in both groups of human fetal hepatocytes (Fig 4E). Overall, these data demonstrates that the inhibition of SIRT1 causes an increase in gluconeogenesis, leading to a higher intracellular glucose concentration in human fetal hepatocytes, through the control of AKT/FOXO1 pathway.

## Discussion

**Fig 5:**
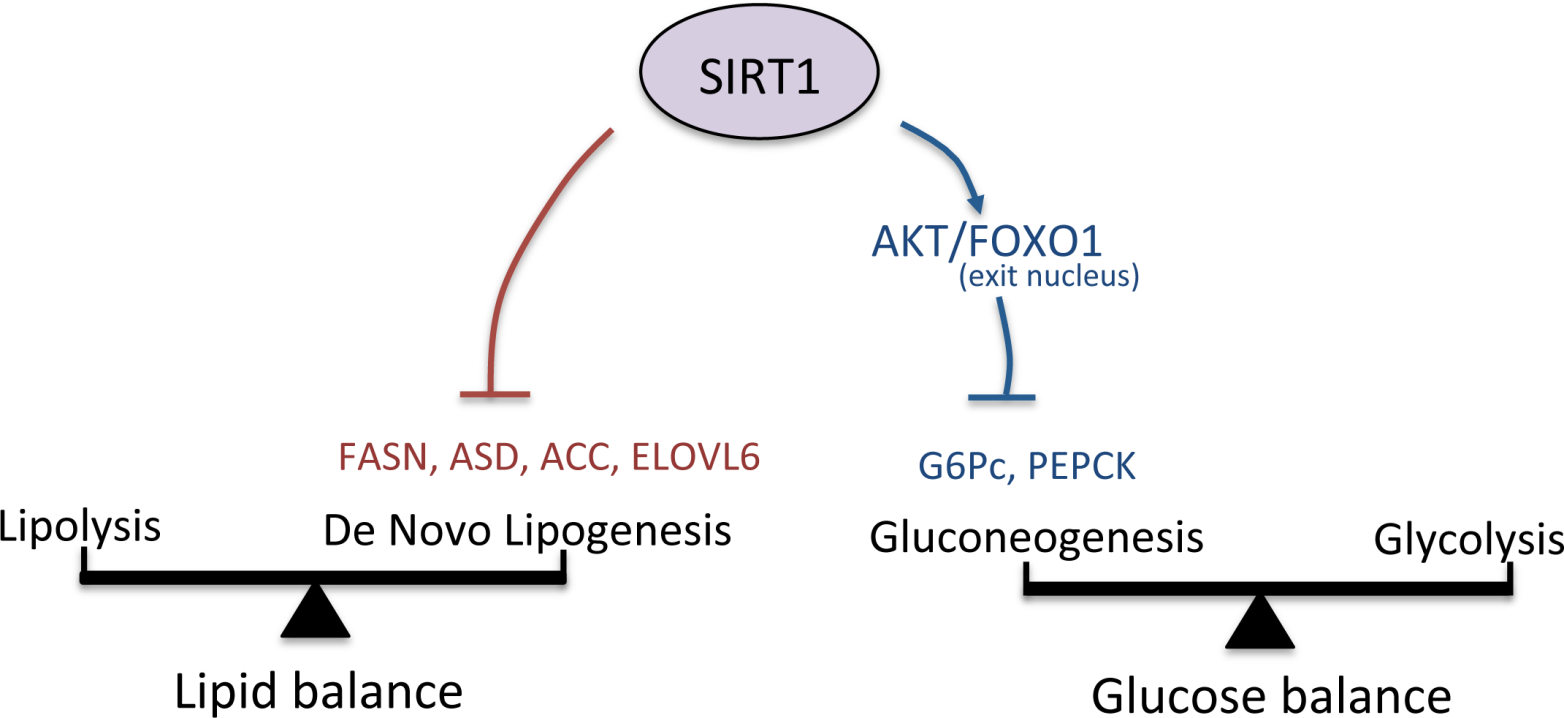
Representation of SIRT1 regulation of lipid and glucose balances in human fetal hepatocytes. In normal conditions, SIRT1 inhibits De Novo Lipogenesis and Gluconeogenesis through the AKT/FOXO1 pathway inside human fetal hepatocytes, keeping a normal balance inside the cells. Upon SIRT1 inhibition, both the lipid and glucose balance will be disrupted, leading to an increase of lipid and carbohydrates levels in human fetal hepatocytes.

In the recent years, researchers have just started exploring and understanding the importance of SIRT1 in metabolism. SIRT1 acts as a metabolic “switch” linked to hepatic steatosis. To the best of our knowledge, this is the first report that showed the implication of SIRT1 in metabolic processes in human fetal hepatocytes. In this study, we observed that the inhibition of SIRT1 using a pharmacological agent for a short period of time leads to a rapid increase in lipid and carbohydrate concentrations. Moreover, we have confirmed a negative regulation of SIRT1 in *de novo* lipogenesis and gluconeogenesis through the AKT/FOXO1 pathway in human fetal hepatocytes. These results are coherent with previously studies on mice carrying a deletion of SIRT1 in the liver and developing liver steatosis. These results also show that human fetal hepatocytes are responsive to SIRT1 signals and emphasize SIRT1 role as a regulator of lipid and glucose balances in the liver (Fig 5). Altogether, this study confirmed the extensive role of SIRT1 in hepatocytes metabolism during development.

Previous studies have already documented an increase in lipogenesis and gluconeogenesis during fetal development [28, 29]. The maternal diet (fructose diet and high fat diet) imposed these metabolic changes on the fetus. It suggests that changes in the maternal diet can provide more nutrients availability, used as energy blocks by the fetus to sustain these metabolic pathways. Similarly, exposure to certain factors (such as a high fat diet) might affect SIRT1 in the liver and induce metabolic changes during development. In the long run (after birth), an increase production of lipids could render these offsprings more susceptible to the development of hepatic steatosis and hence hyperglycemia that is linked to insulin resistance and diabetes [30, 31]. Consequently, more sophisticated models to study human tissues overtime (infants and adults) are needed to address questions regarding the important role of SIRT1 and the molecular mechanistic upstream and downstream.

Our future studies will investigate which signaling pathways under SIRT1 influence the lipid metabolism on these cells. For instance, SIRT1 has been shown to deacetylate Liver X Receptor (LXR), which is a regulator of lipid homeostasis [32]. However, we were not able to detect differences in LXR gene targets: ATP-binding cassette, sub-family A (ABC1), member 1 or Srebp-1c (S2B Fig). This suggests that SIRT1 may acts through another pathway in human fetal hepatocytes. In the other hand, AKT/FOXO1 pathway is a major regulator of glucose production in response to insulin, nevertheless, it has been shown that SIRT1 represses gluconeogenesis during fasting, through CREB-regulated transcription coactivator 2 (CRTC2) deacetylation, leading to its degradation [33]. However, we did not find significant changes in CRTC2 expression when SIRT1 was inhibited (S2C Fig). These results support the main role of AKT/FOXO1 pathway to regulate gluconeogenesis under the control of SIRT1 in human fetal hepatocytes.

Numerous rodent studies have shown that the parental dietary may affect the development of diseases later in life, such as diabetes or obesity, by changing the epigenetic signature of the offspring [34]. Recently, studies performed on animal models reported that maternal high fat diet and obesity resulted in a decrease of SIRT1 mRNA in fetal livers [35, 36], highlighting the role of intrauterine environment on SIRT1 expression. However, animal models do not mimic the pathogenesis of human metabolic diseases and further studies on human tissues will be required to determine what controls SIRT1. It will be interesting to determine whether SIRT1 acts as an adaptive transcriptional response during human development. In this respect, SIRT1 could respond to external environmental cues such as maternal obesity and create the conditions to develop NAFLD. A better understanding of the central position of SIRT1 in hepatic metabolism will help in the comprehension of the metabolic diseases and will provide valuable information to prevent and treat human metabolic abnormalities.

## Supporting Information

S1 Fig Expression of SIRT1 in fetal liver cells. Western blot analysis of SIRT1 in human fetal liver cells (fibroblasts and hepatocytes). A human fetal fibroblasts cell line carrying a shMIR-SIRT1 activated upon doxycycline addition was used to assess the specificity of SIRT1 expression.
(TIF)

S2 Fig Analysis of alternative β-oxidation, lipogenesis and gluconeogenesis related genes. (A) Expression of hepatic ACOX1 and ACADM mRNA measured by qRT- PCR in human fetal hepatocytes exposed to +50uM Sirtinol compared to controls. (B) Expression of hepatic ABCA1 and SREBP-1C mRNA expression measured by qRT- PCR in human fetal hepatocytes exposed to +50uM Sirtinol compared to controls. (n≥7/group; *, P < .05) (C) Western blot analysis for CRCT2 in human fetal hepatocytes exposed to +50uM Sirtinol. GAPDH was used as a control.
(TIF)
