# Supplementary figures and images for: SIRT1 Disruption in Human Fetal Hepatocytes Leads to Increased Accumulation of Glucose and Lipids
Source: PLoS One. 2016 Feb 18;11(2):e0149344. doi: 10.1371/journal.pone.0149344 (PMC4758736; doi:10.1371/journal.pone.0149344)

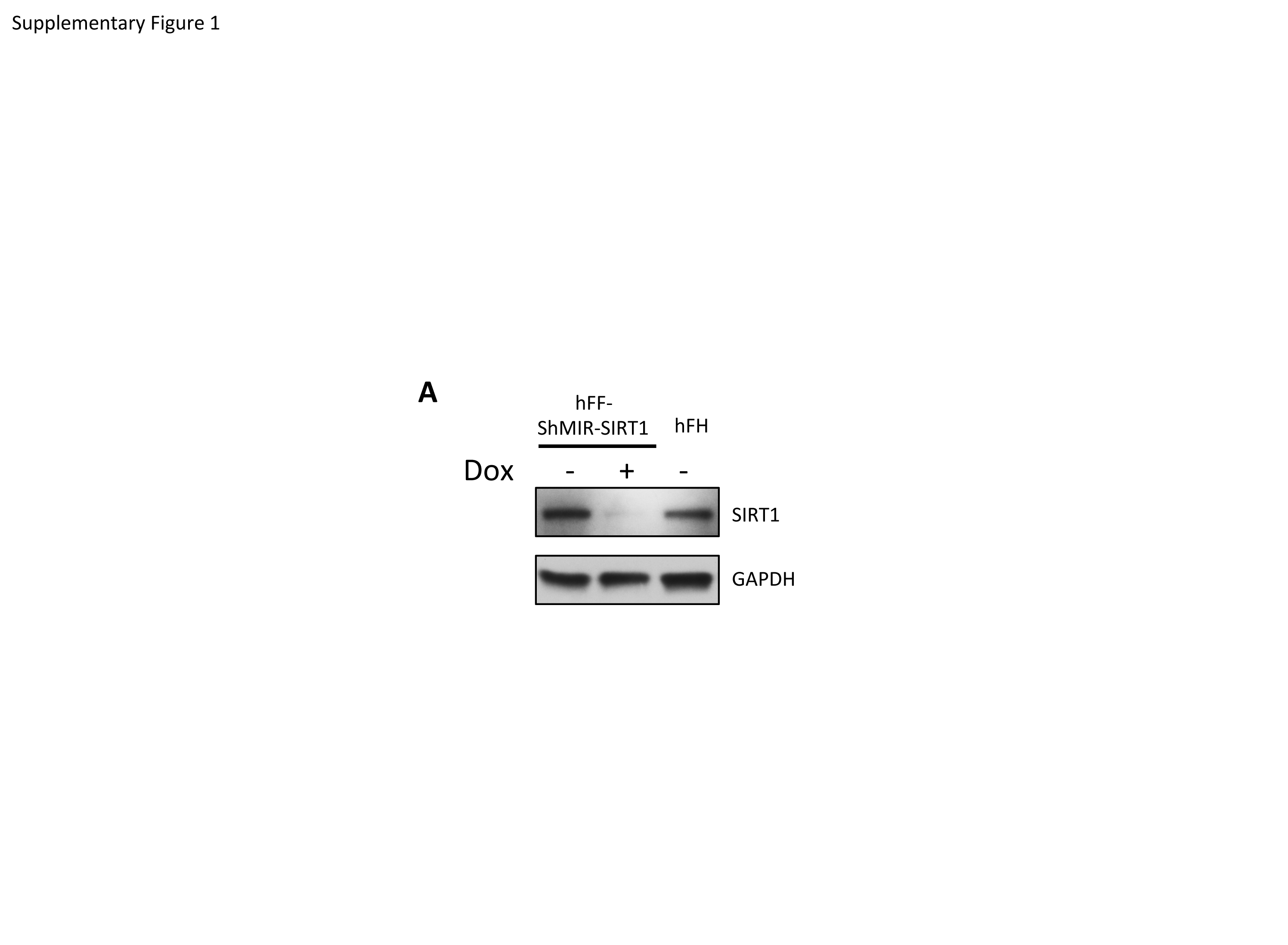

Supplement: S1 Fig — Western blot analysis of SIRT1 in human fetal liver cells (fibroblasts and hepatocytes). A human fetal fibroblasts cell line carrying a shMIR-SIRT1 activated upon doxycycline addition was used to assess the specificity of SIRT1 expression. (TIF) [file pone.0149344.s001.tif]

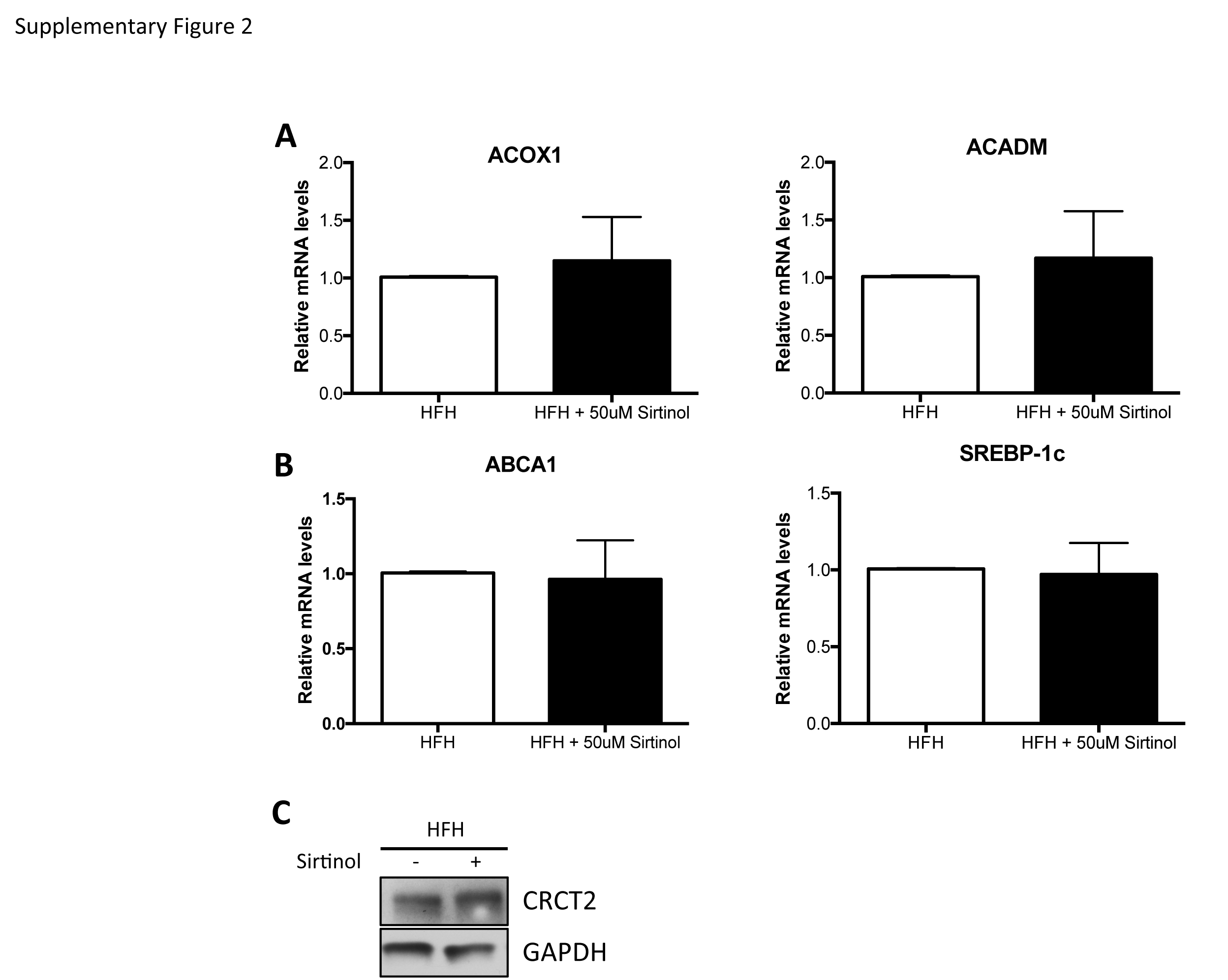

Supplement: S2 Fig — (A) Expression of hepatic ACOX1 and ACADM mRNA measured by qRT- PCR in human fetal hepatocytes exposed to +50uM Sirtinol compared to controls. (B) Expression of hepatic ABCA1 and SREBP-1C mRNA expression measured by qRT- PCR in human fetal hepatocytes exposed to +50uM Sirtinol compared to controls. (n≥7/group; *, P < .05) (C) Western blot analysis for CRCT2 in human fetal hepatocytes exposed to +50uM Sirtinol. GAPDH was used as a control. (TIF) [file pone.0149344.s002.tif]
